# Exercise, Mood, Self-Efficacy, and Social Support as Predictors of Depressive Symptoms in Older Adults: Direct and Interaction Effects

**DOI:** 10.3389/fpsyg.2019.02145

**Published:** 2019-09-19

**Authors:** Kyle J. Miller, Christopher Mesagno, Suzanne McLaren, Fergal Grace, Mark Yates, Rapson Gomez

**Affiliations:** ^1^School of Health and Life Sciences, Federation University, Ballarat, VIC, Australia; ^2^Faculty of Health, School of Medicine, Ballarat Health Services, Deakin University, Ballarat, VIC, Australia

**Keywords:** elderly, seniors, physical activity, depression, hierarchical regression, moderation, interaction

## Abstract

**Background:**

Depression is a chronic condition that affects up to 15% of older adults. The healthogenic effects of regular exercise are well established, but it is still unclear which exercise-related variables characterise the antidepressant effects of exercise. Thus, the purpose of this study was to examine the extent to which exercise-related variables (exercise behaviour, exercise-induced mood, exercise self-efficacy, and social support) can predict depressive symptoms in a cohort of community-dwelling older adults.

**Methods:**

This study employed a cross-sectional analysis of questionnaire data from a sample of 586 community-dwelling older Australians aged 65 to 96 years old. Participants completed the Center for Epidemiologic Studies Depression Scale, modified CHAMPS Physical Activity Questionnaire for Older Adults, Four-Dimension Mood Scale, Self-Efficacy for Exercise Scale, and Social Provisions Scale – Short Form. Bivariate correlations were performed, and hierarchical multiple regression was subsequently used to test the regression model.

**Results:**

Exercise behaviour, exercise-induced mood, exercise self-efficacy, and social support were all negatively associated with depressive symptoms (*r* = −0.20 to −0.56). When the variables were entered as predictors into the hierarchical multiple regression model, social support was the strongest predictor of depressive symptoms (β = −0.42), followed by exercise-induced mood (β = −0.23), and exercise self-efficacy (β = −0.07). Exercise behaviour did not explain any additional variance in depressive symptoms. A modest interaction effect was also observed between exercise-induced mood and social support.

**Conclusion:**

These findings indicate that social support is the strongest predictor of depressive symptomology in community-dwelling older adults, particularly when combined with positive exercise-induced mood states. When addressing the needs of older adults at risk of depression, healthcare professionals should consider the implementation of exercise programmes that are likely to benefit older adults by improving mood, enhancing self-efficacy, and building social support.

## Introduction

The proportion of older adults aged 65 years and over in the Australian population is gradually rising, leading to an ageing population (Australian Bureau of Statistics, 2016). Ageing is associated with a greater prevalence of long-term health problems, including significantly higher rates of comorbid mental health problems within the older demographic (Barnett et al., 2012; Volkert et al., 2013). Depression is one of the most prevalent mental health problems in older adulthood (Argyropoulos et al., 2012), with estimates of 10–15% of community-dwelling older adults aged 65 years and over experiencing depressive symptoms at any given time (Pirkis et al., 2009; Luppa et al., 2012; Laborde et al., 2015). Depressive symptoms can include a persistent sadness, irritable or anxious mood, reduced concentration and attention, low energy levels or fatigue, low self-esteem or self-confidence, feelings of hopelessness, sleep or appetite problems, loss of interest or pleasure in activities that used to be enjoyable, or difficulty in carrying out usual work or social activities (American Psychiatric Association, 2013).

To date, researchers have largely focused on interventions that can delay the ageing process, and in doing so, encourage a healthspan consistent with prolonged positive mental health in later life. Physical exercise is commonly integrated into daily living to build social support and enhance quality of life, resulting in a significant increase to longevity (Gremeaux et al., 2012; Vagetti et al., 2014). Exercise refers to structured and repetitive concentric, eccentric, or isometric muscular activity performed to improve or maintain one or more aspects of physical or psychological health (Caspersen et al., 1985; Winter and Fowler, 2009; Ceria-Ulep et al., 2011). Exercise has been identified as a key protective factor against depression during older adulthood (see Rhyner and Watts, 2016 for a review), and may be used in addition to traditional treatments, such as antidepressant drug therapy, to further reduce depressive symptoms (Mura et al., 2014; Belvederi Murri et al., 2019).

Clarity in geriatric depression research has been widely discussed during the past two decades (Blazer, 1997), but there remains a pervasive inconsistency of research outcomes, and many common misconceptions (Haigh et al., 2018). In part, this is likely due to the weight of scientific inquiry being heavily directed toward younger and middle-aged adults, relative to older populations (evidenced by brief literature search; see Supplementary File S1). Moreover, the geriatric literature remains focused on single isolated factors which forgoes the complexity of important interactions. Three key underlying psychosocial mechanisms predominate in understanding the relationship between exercise and depression, including a distraction from negative mood states, enhanced self-efficacy, and increased social support (see Paluska and Schwenk, 2000 for a theoretical review).

The earliest explanation for the depression-reducing effects of exercise is the distraction hypothesis, which is based on the theory that a simple diversion from a painful stimulus can lead to an improved mood state following physical exercise (Festinger and Maccoby, 1964). This was initially demonstrated in an intervention study (Bahrke and Morgan, 1978) whereby 75 adult participants engaged in 20 min of treadmill exercise, general meditation, or reading in a sound-dampened chamber. Bahrke and Morgan (1978) reported that participants had a better post-intervention affect in all forms of activity, concluding that exercise, as well as other activities, provide a valuable distraction from the stress of daily living, anxiety, and depression. Evidence from a meta-analysis conducted by Arent et al. (2000) has further supported the mood-inducing effect of exercise in older adults. A quantitative analysis of 32 studies found that chronic exercise leads to significantly more positive affect (Hedges’ *g* = 0.33) and less negative affect (Hedges’ *g* = 0.35) compared to control conditions, indicating that exercise has acute effects on negative dispositions (Arent et al., 2000).

According to self-efficacy theory, an individual’s depressive symptoms may also become less severe should their self-efficacy become increased (Bandura, 1977, 1997). Exercise self-efficacy is defined as one’s beliefs about the capability to successfully engage in incremental bouts of physical activity (Blacklock et al., 2010). When older adults engage in regular exercise, it provides them with a greater sense of mastery and personal control over their environment, which they can then transfer onto other everyday tasks to cope with stress and depression. Preliminary evidence has identified self-efficacy as an independent mechanism for the antidepressant effects of exercise in adult samples (Craft, 2005; Ryan, 2008; Annesi, 2012). Moreover, a recent cross-sectional study of 42 older adults revealed that depressive symptoms were independently and negatively associated with exercise self-efficacy (Byrne and Horgan, 2018). Since the size of this sample was underpowered, however, further investigation is necessary to ascertain the extent to which exercise self-efficacy independently predicts depressive symptoms.

Affective responses and efficacy beliefs have been proposed to form two distinct, yet interrelated, influences on mental health (Bandura, 1997). A randomised controlled trial on a small cohort of 12 clinically depressed adults (*M*_*age*_ = 36.6 years) initially reported that acute exercise aimed at enhancing self-efficacy had significant improvements on self-reported mood (Bodin and Martinsen, 2004). Subsequently, Pickett et al. (2012) employed a cross sectional study of 164 moderately depressed adults (*M*_*age*_ = 30 years), theorising that self-efficacy mediates the exercise-depression relationship through positive affect. A single longitudinal study has supported this tenet, whereby exercise-induced mood interacts with self-efficacy in depressed adults (White et al., 2009). To date, few studies have examined the independent and interactive effects of exercise-induced mood and self-efficacy on depressive symptoms in older cohorts, and therefore, further research is needed to establish these patterns in older age.

The capacity for exercise to build social support networks and concomitant positive effects on mood and depression during older adulthood is widely accepted (McAuley et al., 2000; Makino et al., 2015). Social support refers to the helpful resources an individual perceives available to them in the context of both formal and informal relationships (Gottlieb and Bergen, 2010). For older adults, physical exercise allows opportunities to engage in challenges, adjust to retirement, and maintain personal relationships and endeavours (Bengtson et al., 2009). Consequently, older individuals gain a sense of social connectedness by developing new and diverse shared experiences (Lindsay-Smith et al., 2018), resulting in an improved ability to cope with stress and depression (Glass et al., 2006; Cornwell and Waite, 2009). Indeed, a cross-sectional study involving 583 community-dwelling older adults revealed that both exercise behaviour and social support independently predicted depressive symptoms (McHugh and Lawlor, 2012).

Despite the wealth of evidence for the benefits of social interaction and support on depressive symptoms in older cohorts (Gariépy et al., 2016), few studies have investigated whether social support interacts with exercise self-efficacy, and/or mood to further predict depressive symptoms. Preliminary longitudinal research has shown that self-efficacy reduces depression directly as well as interacting indirectly through social support (Holahan and Holahan, 1987). McAuley et al. (2000) found that a social, group-based exercise predicted stronger changes in affective states (i.e., positive well-being, lower psychological distress, and fewer feelings of fatigue) than individual exercise in a group of older adults (*n* = 80). When self-efficacy was included in the model, more positive and less negative mood states occurred, but these mood states were dependent on the intensity and social conditions of exercise (McAuley et al., 2000). Interestingly, these findings outline potential interaction effects between social support and exercise-induced mood and/or self-efficacy on depressive symptoms, which are worthy of examination in a larger participant sample.

Because researchers have examined depressive symptoms and their associated predictors in isolation (i.e., exercise behaviour, exercise-induced mood, exercise self-efficacy, and social support), there is currently limited benchmarking of predictors within the same analysis, accounting for overlapping variance and potential interaction effects between two predictor variables. Moreover, existing studies have predominately tested these associations in younger adult cohorts without consideration of age-related changes, such as deficits in health status and/or physical functioning (Barry et al., 2011; Nyunt et al., 2012).

This study extends previous scientific enquiry in several advantageous ways: (1) exercise behaviour, exercise-induced mood, exercise self-efficacy, and social support are tested in the same regression model, allowing effects to be quantified according to how much unique variance can be explained by a specific predictor variable while controlling for all other variables, (2) interaction effects are tested between predictor variables, which can assist in identifying potential underlying synergistic effects between two seemingly independent predictors, and (3) this study employs an adequately powered sample (*n* > 300), thus reducing the opportunity for Type II errors.

The purpose of this study was to investigate the extent to which exercise-related variables (exercise behaviour, exercise-induced mood, exercise self-efficacy, and social support) predict depressive symptoms in a sample of community-dwelling older adults (>65 years) by employing hierarchical multiple regression techniques. A regression model was hypothesised whereby exercise behaviour, exercise-induced mood, exercise self-efficacy, and social support were used to predict depressive symptoms in older adults. This model was used to compare the strength of prediction estimates, controlling for health status, physical functioning, age, gender, and other known predictor variables. Two-way interaction effects between exercise-induced mood, exercise self-efficacy, and social support in the model were further explored.

## Materials and Methods

### Participants

Ethical approval was obtained from the Australia Human Research Ethics Committee prior to data collection. The study included a sample of 586 community-dwelling older Australians aged over 65 years, recruited from retirement villages, senior citizen centers, community groups, bowling clubs, and fitness centers. The sample consisted of 172 males and 414 females, with ages ranging from 65 to 96 years (*M* = 72.47, *SD* = 6.22). Full participant demographics can be found in Table 1. In general, participants had a secondary school qualification or higher, lived with a partner or alone, were married, and currently retired. Most participants reported good health or better, with no physical limitations or only minor limitations.

**TABLE 1:** Participant descriptive data.

**Variable**	**Total (*n* = 586)**	**Electronic (*n* = 435)**	**Paper (*n* = 151)**	**Between-groups**
Age (years)	72.47 ± 6.22	71.43 ± 5.38	75.47 ± 7.41	–7.16^∗∗∗^
Body weight (kg)	72.58 ± 15.52	72.11 ± 15.22	73.93 ± 16.33	–1.23
Exercise behaviour (calories/week)	1307 ± 1315	1306 ± 1245	1314 ± 1527	–0.03
Females, *n* (%)	414 (70.65)	309 (71.03)	105 (69.54)	0.12
CES-D ≥ 16, *n* (%)	129 (22.01)	91 (20.92)	38 (25.17)	1.18
Pet owner, *n* (%)	196 (33.45)	152 (34.94)	44 (29.33)	1.58
**Health status, *n* (%)**				
Excellent	89 (15.19)	72 (16.55)	17 (11.26)	9.20
Very good	240 (40.96)	184 (42.23)	56 (39.07)	
Good	181 (30.89)	132 (30.35)	49 (32.45)	
Fair	67 (11.43)	41 (9.43)	26 (17.22)	
Poor	9 (1.54)	6 (1.38)	3 (1.99)	
**Physical functioning, *n* (%)**				
Not limited	264 (45.05)	207 (47.59)	57 (37.75)	4.66
A little limited	269 (45.90)	192 (44.14)	77 (50.99)	
Very limited	53 (9.04)	36 (8.28)	17 (11.26)	
Living arrangements, *n* (%)				
Partner	344 (58.70)	267 (61.38)	77 (50.99)	5.50
Family members	34 (5.80)	22 (5.06)	12 (7.95)	
Friends	4 (0.68)	3 (0.69)	1 (0.66)	
Alone	204 (34.81)	143 (32.87)	61 (40.40)	
**Relationship status, *n* (%)**				
Partner	43 (7.34)	34 (7.82)	9 (6.00)	18.82^∗∗^
Single	37 (6.31)	30 (6.90)	7 (4.67)	
Married	313 (53.41)	242 (55.63)	71 (47.33)	
Separated/divorced	85 (14.51)	67 (15.40)	18 (12.00)	
Widowed	107 (18.26)	62 (14.25)	45 (30.00)	
**Education, *n* (%)**				
Primary	17 (2.90)	3 (0.69)	14 (9.33)	87.34^∗∗∗^
Secondary	174 (29.69)	100 (22.99)	74 (49.33)	
TAFE/trade cert	119 (20.31)	90 (20.69)	29 (19.33)	
Undergraduate	105 (17.92)	84 (19.31)	21 (14.00)	
Postgraduate	170 (29.01)	158 (36.32)	12 (8.00)	
**Employment status, *n* (%)**				
Full-time	5 (0.85)	4 (0.92)	1 (0.66)	21.26^∗∗∗^
Part-time/casual	51 (8.70)	46 (10.57)	5 (3.31)	
Homemaker	13 (2.22)	4 (0.92)	9 (6.00)	
Retired	513 (87.54)	377 (86.67)	136 (90.07)	
Unemployed	4 (0.68)	4 (0.92)	0 (0.00)	

*Descriptive data are reported as means and standard deviations unless otherwise stated. Between-groups test statistic was *t* for continuous values and χ^2^ for categorical values. Missing data for body weight (*n* = 3), pet owner (*n* = 1), relationship status (*n* = 1), and education (*n* = 1). ^∗^*p* < 0.05; ^∗∗^*p* < 0.01; ^∗∗∗^*p* < 0.001.*

### Measures

Data were collected using a battery of questionnaires, including a demographics questionnaire, Center for Epidemiologic Studies Depression Scale (CES-D; Radloff, 1977), modified CHAMPS Physical Activity Questionnaire for Older Adults (Stewart et al., 2001), Four-Dimension Mood Scale (4DMS; Huelsman et al., 1998), Self-Efficacy for Exercise (SEE) Scale (Resnick and Jenkins, 2000), and Social Provisions Scale – Short Form (SPS-10; Cutrona and Russell, 1987).

#### Demographics Questionnaire

Participants were asked to complete a brief demographic questionnaire requesting background information on age, gender, weight, living arrangement, education, ethnicity, employment status, relationship status, and ownership of pets. Two items from the 36-item Short-Form Health Survey (SF-36; Ware and Sherbourne, 1992) were also adapted and included on the demographic questionnaire to assess health status (i.e., *in general, how would you rate your health?*) and physical functioning (i.e., *does your health limit you in exercise-related activities?*).

#### Center for Epidemiologic Studies Depression Scale (CES-D)

The Center for Epidemiologic Studies Depression Scale is a 20-item self-report measure of depressive symptom severity (Radloff, 1977), which is suitable for use within older adult samples (Górkiewicz and Chmiel, 2015). The CES-D reflects four distinct factors: depressive affect, somatic symptoms, interpersonal distress, and positive affect. Participants were instructed to report how often they experience particular depressive symptoms (e.g., *I felt lonely*) over the preceding 4 weeks on a 4-point Likert scale from 0 (*rarely or none of the time - less than 1 day*) to 3 (*most or all of the time – 5 to 7 days*), with the four positive affect items reverse scored.

Past studies have found high internal consistency for the total CES-D in older adults, with alpha coefficients above 0.80 (Ros et al., 2011; Górkiewicz and Chmiel, 2015). A Cronbach’s alpha of 0.86 was calculated for the current sample. The CES-D has been successfully used to identify clinical depression (Ros et al., 2011), as well as being concurrently validated with subscales of depression (*r* = 0.58), and suicide ideation (*r* = 0.47) on the General Health Questionnaire in older adults (Gomez and McLaren, 2015).

#### Modified CHAMPS Physical Activity Questionnaire for Older Adults

The CHAMPS is a 28-item questionnaire designed to measure the caloric expenditure of physical activity during older adulthood, which takes into account the intensity, frequency, and duration of activity, as well as correcting for differences in body weight (Stewart et al., 2001). For the current study, a modified CHAMPS comprising of 20 items was used to measure exercise behaviour (Resnicow et al., 2003). Exercise behaviour was operationally defined as weekly caloric expenditure in intentional sport and recreational activities that are not part of daily functioning. Participants were instructed to report how many times per week and total hours in a typical week they have engaged in each exercise-related activity (e.g., *do water exercises*) over the preceding 4 weeks.

The CHAMPS correlated highly with two commonly used physical activity measures, including the Physical Activity Questionnaire Physical Activity Survey for the Elderly (*r* = 0.64) and Yale Physical Activity Survey (*r* = 0.68), and test-retest reliability was 0.76 over a 2-week period (see Harada et al., 2001 for measure comparisons and rationale). The CHAMPS also has extensive construct validity with measures of lower body functioning and endurance (0.15 to 0.28; Stewart et al., 2001), physical health (*r* = 0.14 to 0.32; Cyarto et al., 2006), and pedometer step count (*r* = 0.21 to 0.57; Giles and Marshall, 2009).

#### Four-Dimension Mood Scale (4DMS)

The Four-Dimension Mood Scale is a 20-item questionnaire designed to measure four dimensions of dispositional mood: positive energy, negative arousal, tiredness, and relaxation (Huelsman et al., 1998). Participants were instructed to indicate to what extent each adjective item reflects the way they generally feel after exercise (e.g., *energetic*) on a 5-point Likert scale from 1 (*very slightly or not at all*) to 5 (*extremely*).

The Four-Dimension Mood Scale was selected because it was conceptualised with a theoretical understanding of affect and mood, uses exercise-relevant mood adjectives, has less floor and ceiling effects, avoids dimension loading problems by not using reverse worded items, and involved sufficient confirmatory factor analysis procedures during item selection and questionnaire validation (Boyle et al., 2015; see Ekkekakis, 2013 for measure comparisons and rationale). Moreover, the 4DMS is more sensitive to the effects of physical exercise than other similar mood measures (Gregg and Shepherd, 2009). Cronbach’s alpha values ranged between 0.87 and 0.93 in a sample of adults (*M*_*age*_ = 42.6; Huelsman et al., 1998). The current study reported a Cronbach coefficient of 0.87. Concurrent validity was found between the 4DMS and similar mood measures, including the Activation-Deactivation Adjective Check List (*r* = 0.65 and 0.45), Job Affect Scale (*r* = 0.82 and 0.75), and Positive and Negative Affect Schedule (*r* = 0.62 and 0.68; Huelsman et al., 2003).

#### Self-Efficacy for Exercise (SEE) Scale

The nine-item Self-Efficacy for Exercise (SEE) Scale (Resnick and Jenkins, 2000) assesses one’s self-efficacy expectations in their ability to continue exercising in the face of perceived barriers, which has been developed for use in older populations (see McAuley et al., 2013 for measure comparisons and rationale). Participants were instructed to report their confidence in engaging in exercise 3 times a week for 20 min if faced with a barrier (e.g., *if you were bored by the programme or activity*) from 0 (*not confident*) to 10 (*very confident*).

Resnick et al. (2004) reported excellent internal consistency in older adults (α = 0.89 and 0.90). Likewise, a Cronbach’s alpha value of 0.90 was calculated in the current sample. The SEE reflects good construct validity, correlating with outcome expectations (*r* = 0.39), physical functioning (*r* = 0.31), general health perceptions (*r* = 0.23), mental health (*r* = 0.24) regular exercise (*r* = 0.29), and recreational activity (*r* = 0.17; Resnick et al., 2006).

#### Social Provisions Scale – Short Form (SPS-10)

The Social Provisions Scale – Short Form-10 is a shortened, 10-item version of the original 24-item questionnaire (Cutrona and Russell, 1987), which was designed to measure the degree to which an individual’s social relationships provide six dimensions of social support: attachment, social integration, reassurance of worth, reliable alliance, guidance, and opportunity for nurturance (Russell et al., 1984). Confirmatory factor analysis indicated that the six subscales reflect six first-order factors and a single second-order factor structure (Cutrona and Russell, 1987). The opportunity for nurturance subscale was excluded in the shortened measure because it reflects a dimension of social support that is provided to others rather than received. Participants were instructed to rate the strength of their agreement for each statement (e.g., *there are people who enjoy the same social activities as I do*) on a 4-point Likert scale from 1 (*strongly disagree*) to 4 (*strongly agree*). Each of the five subscales comprised of two items, with one positively worded and one negatively worded (reverse scored) item, for a total of 10 items.

The Social Provisions Scale – Short Form-10 was selected over the original SPS because it is more manageable for older adults while maintaining strong psychometrical properties (see Gottlieb and Bergen, 2010 for measure comparisons and rationale). Internal consistency for the SPS-10 was 0.75 in a sample of older adults (Randall et al., 2010). The Cronbach’s alpha value was 0.83 in the current sample. Total scores on the SPS correlated with life dissatisfaction, loneliness, and depression (*r* = −0.28 to −0.31), indicating good construct validity (Cutrona et al., 1984, unpublished). Moreover, the social integration (*r* = 0.14) and reliable alliance (*r* = −0.25) subscales were predictive of follow-up depression (Russell and Cutrona, 1991).

### Procedures

Interested participants received a questionnaire package, which included a plain language statement and the battery of questionnaires. For participants who elected to complete the package in their own time, a reply-paid envelope was also supplied. An electronic version of the questionnaire using the SurveyMonkey online software was available for those who were recruited indirectly via social media or community advertisements. The questionnaire package took approximately 20 min to complete and measures were arranged in a randomised order to prevent any order effects.

### Power Analysis and Sample Size

*A priori* sample size calculation was used to determine the approximate lower bound sample size needed to detect the specified effects (Soper, 2019). The calculation was made based on a conservative effect size (Cohen’s *f*^2^ = 0.05) and the number of predictor variables in the model (*m* = 13), desired statistical power level (π = 0.8), and significance level (*p* = 0.05). The required sample size for the regression model was calculated to be a minimum of 368 participants to detect a small effect size.

### Statistical Analysis

All statistical procedures were performed using STATA/SE 15.1 (StataCorp, 2017). Missing data were imputed using multiple imputation (using the MI IMPUTE command in Stata) with a recommended 20 imputed datasets (Sterne et al., 2009). Sensitivity analyses using independent *t*-tests were performed to compare variables between complete and imputed cases. No significant differences were found for any imputed variable, and therefore, missing participant data were assumed to be missing at random. Normality of the data was confirmed, then parametric statistics were used. Notably, social support was negatively skewed (see Supplementary File S1 for a histogram), however, subsequent transformations resulted in greater skewness than the untransformed data. Independent *t*-tests and χ^2^ tests were also used to compare demographic and outcome statistics between paper and electronic questionnaires.

Descriptive statistics are reported as means (*M*), standard deviations (*SD*), and Pearson product-moment correlation coefficients (*r*). Hierarchical multiple regression was used to test the regression model and interaction effects. Predictor variables were standardised by centring the scores at the mean. Control variables for health status, physical functioning, age, and gender were entered in Step 1 of the regression model, then the hypothesised predictor variables (i.e., exercise behaviour, exercise-induced mood, exercise self-efficacy, and social support) were entered in Step 2. Interaction terms (predictor × predictor) were entered in Step 3 of the model to test the interactions between two predictor variables. Beta coefficients (β) provided standardised comparisons between predictor variables and squared semi-partial correlation coefficients (*sr*^2^) indicated the proportion of unique effect contributed by each predictor variable. Confidence intervals (95% CI), Cohen’s *f*-squared (*f*^2^), and *p*-values were used to examine significant effects in the regression model. Cohen’s (1988) guidelines were used to evaluate the strength of the *f*^2^ effect size, whereby 0.02 is considered ‘small,’ 0.15 is considered ‘moderate’ and 0.35 is considered ‘large.’ Statistical significance was achieved with criterion *p* < 0.05.

## Results

### Descriptive Statistics

Data from a total of 586 participants were included in the analysis. A total of 230 paper questionnaire packages were distributed, with 151 usable packages being returned (65.65% compliance rate). An additional 489 electronic questionnaire packages were completed online, with 435 usable packages providing an 88.96% compliance rate. In total, 586 out of 719 questionnaires were completed with a compliance rate of 81.50%.

Demographic and outcome statistics between paper and electronic questionnaires were statistically compared using independent *t*-tests and χ^2^ tests, which can be found in Table 1. Those who completed the electronic questionnaire package were generally younger (*M*_*diff*_ = 4.04 years; *t* = −7.16; *p* < 0.001) and had a higher level of education (χ^2^ = 87.34; *p* < 0.001). Significant differences were also observed for relationship status (χ^2^ = 18.82; *p* < 0.01) and employment status (χ^2^ = 21.26; *p* < 0.001). These differences were unremarkable, and therefore, data were collapsed into a single dataset.

Full descriptive statistics and bivariate correlations (Pearson product-moment correlation coefficients) are characterised in Table 2. Briefly, depressive symptoms were significantly and negatively associated with all hypothesised predictor variables, including (1) exercise behaviour, (2) exercise-induced mood, (3) exercise self-efficacy, and (4) social support. Both health status and physical functioning demonstrated significant relationships with exercise behaviour, exercise-induced mood, exercise self-efficacy, and social support, and were subsequently included in the subsequent regression model.

**TABLE 2:** Means, standard deviations, and intercorrelations among study variables.

**Variable**	**M ± SD**	**1**	**2**	**3**	**4**	**5**	**6**	**7**	**8**
1. Age	72.47 ± 6.22	1							
2. Health status	3.57 ± 0.93	–0.06	1						
3. Physical functioning	2.36 ± 0.64	–0.11^∗∗^	0.52^∗∗∗^	1					
4. Depressive symptoms	10.73 ± 8.38	–0.07	–0.43^∗∗∗^	–0.34^∗∗∗^	1				
5. Exercise (calories/week)	1307 ± 1315	–0.17^∗∗∗^	0.32^∗∗∗^	0.28^∗∗∗^	–0.20^∗∗∗^	1			
6. Exercise-induced mood	76.78 ± 9.84	–0.08	0.42^∗∗∗^	0.40^∗∗∗^	–0.50^∗∗∗^	0.33^∗∗∗^	1		
7. Exercise self-efficacy	51.14 ± 21.87	−0.10^∗^	0.30^∗∗∗^	0.24^∗∗∗^	–0.28^∗∗∗^	0.44^∗∗∗^	0.43^∗∗∗^	1	
8. Social support	33.79 ± 4.61	0.02	0.26^∗∗∗^	0.16^∗∗∗^	–0.56^∗∗∗^	0.09^∗^	0.33^∗∗∗^	0.11^∗^	1

*Higher mean values reflect a high level on the given variable. ^∗^*p* < 0.05; ^∗∗^*p* < 0.01; ^∗∗∗^*p* < 0.001.*

### The Regression Model

The regression model was tested using hierarchical multiple regression to compare the strength of prediction estimates from exercise-related variables (exercise behaviour, exercise-induced mood, exercise self-efficacy, and social support) on depressive symptoms, after controlling for health status, physical functioning, age, and gender (see Table 3). The four control variables were entered at Step 1 of the analysis, accounting for a significant 23.80% of the variance in depressive symptoms. At Step 2, the four predictor variables were entered in the regression analysis, which accounted for a total of 49.83% of the variance in the model as a whole. The addition of the predictor variables account for an additional 26.03% variance in depressive symptoms, Δ*R*^2^ = 26.03, *F*(4,573) = 74.33, *p* < 0.001. In the final model, social support (*sr*^2^ = 0.154), exercise-induced mood (*sr*^2^ = 0.032), and exercise self-efficacy (*sr*^2^ = 0.004) were significant and unique predictors in the combined effect (*f*^2^ = 0.993). According to Cohen’s (1988) conventions, this demonstrates of a large effect size.

**TABLE 3:** Hierarchical multiple regression analysis on depressive symptoms.

**Variable**	**β**	**SE**	***t***	***R*^2^**	**Δ*R*^2^**	***F*(df)**	**VIF**
**Step 1 constant**		4.54	5.93^∗∗∗^	0.24	0.24	22.53^∗∗∗^ (8, 577)	
Health status							
Fair	0.10	2.66	1.02				7.80
Good	–0.10	2.65	–0.71				16.29
Very good	–0.35	2.67	−2.22^∗^				18.94
Excellent	–0.29	2.77	−2.43^∗^				10.96
Physical functioning							
Limited a little	–0.21	1.22	–2.83^∗∗^				4.18
Not limited at all	–0.31	1.30	–4.00^∗∗∗^				4.85
Age	–0.10	0.05	–2.61^∗∗^				1.08
Gender	0.07	0.68	1.89				1.06
**Step 2 constant**		4.39	14.69^∗∗∗^	0.50	0.26	47.43^∗∗∗^ (12, 573)	
Exercise (calories/week)	0.01	0.02	0.37				1.38
Exercise-induced mood	–0.23	0.03	–6.03^∗∗∗^				1.62
Exercise self-efficacy	–0.07	0.01	−2.09^∗^				1.42
Social support	–0.42	0.06	–13.26^∗∗∗^				1.17
**Step 3 constant**		12.46	8.15^∗∗∗^	0.51	0.01	45.25^∗∗∗^ (13, 572)	
Mood × social support	0.98	0.00	3.17^∗∗^				

*VIF, variance inflation factor. ^∗^*p* < 0.05; ^∗∗^*p* < 0.01; ^∗∗∗^*p* < 0.001.*

**FIGURE 1:**
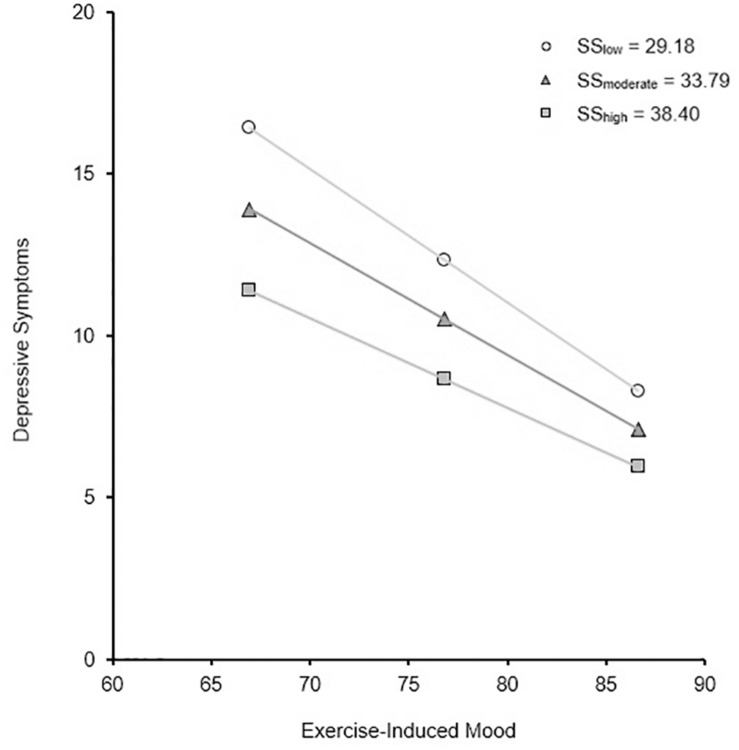
Simple slopes of the relationship between depressive symptoms and exercise-induced mood for low (*M* – 1*SD*), moderate (*M*), and high (*M* + 1*SD*) levels of social support in community-dwelling older adults aged 65–96 years (*n* = 586).

Two-way interaction terms between exercise-induced mood, exercise self-efficacy, and social support were entered independently into Step 3 of the model using an interaction variable (predictor × predictor). There were no interaction effects between exercise-induced mood × exercise self-efficacy (β = 0.19, *t* = −0.77, *SE* = 0.00, *p* = 0.44) nor exercise self-efficacy × social support (β = 0.34, *t* = 1.57, *SE* = 0.00, *p* = 0.12). When exercise-induced mood × social support was entered into the model, a modest interaction effect persisted (β = 0.98, *t* = 3.17, *SE* = 0.00, *p* < 0.01). Exercise-induced mood was significantly related to depressive symptoms which was moderated by social support (see Figure 1).

## Discussion

The main findings from the study demonstrated that social support is the strongest criterion predictor of depressive symptoms, and that exercise self-efficacy and exercise-induced mood also accounted for moderate independent variance within the model. In contrast, exercise behaviour (calories per week) does not provide any additional value to the prediction of depressive symptoms in community-dwelling adults aged 65 years or older.

### Theoretical Implications of the Current Findings

Exercise is known to enhance mood, leading to more positive mood states and a reduction in depression (White et al., 2009; Pickett et al., 2012; Rhyner and Watts, 2016). The current study supports this evidence, indicating that exercise-induced mood can predict lower depressive symptoms in older adults with moderate accuracy. Current theoretical substantiation for this phenomenon is that exercise participation provides a distraction from stress and depression by inducing a positive mood effect (Festinger and Maccoby, 1964). Since affective states are usually short-lived, it is likely that exercise-induced mood reflects acute changes in depressive symptoms. This theory is predicated on the assumption that exercise only promotes positive mood states. If this model holds true, an increased emphasis on the positive experiences of exercise is more likely to achieve the desired effect of reducing depressive symptoms. As with most interventions, however, there are also a proportion of non-respondents to the treatment effect. For those who do not respond to physical exercise (i.e., those who experience negative mood states during exercise such as negative arousal and/or tiredness), emphasis should be placed on other activities that cause a distraction from negative dispositions instead.

The present findings also identify self-efficacy to be an independent predictor of depressive symptoms, albeit at a smaller margin than previously estimated (Craft, 2005; Ryan, 2008; Annesi, 2012; Byrne and Horgan, 2018). We rationalise this to be a consequence of the current regression model because it was able to control for the other predictors and potential confounding variables, estimating an effect size closer to the true effect. Despite a weaker association than exercise-induced mood and social support, these findings indicate that self-efficacy beliefs deriving from regular exercise can predict changes in depressive symptomology. It is therefore conceivable that encouraging older adults to engage in more challenging types of exercise may build the confidence in their ability to accomplish everyday tasks and cope with depression.

In agreement with previous research (McHugh and Lawlor, 2012; Gariépy et al., 2016), these data identify social support to be the strongest predictor of depressive symptoms, and to further interact with mood in strengthening this regression model. Since exercise can be used to maintain personal relationships and increase feelings of social connectedness (Bengtson et al., 2009; Lindsay-Smith et al., 2018), it is likely that group-based exercise can effectively increase the ability to cope with stress and depression (Glass et al., 2006; Cornwell and Waite, 2009). Therefore, interventions that focus on building social support through exercise may be implemented in older at-risk cohorts to strengthen resilience against depressive symptoms.

Lastly, an interaction effect was observed between exercise-induced mood and social support on depressive symptoms, reflecting a monotonic interaction. This indicates that when social support is low, positive mood states from exercise have a stronger association with depressive symptoms, and vice versa. Thus, this interaction can be interpreted in one of two ways: (1) those with lower social support respond better to positive mood states, or (2) those with more negative mood states respond better to social support. Although this interaction was modest, social support together with the mood-enhancing benefits of exercise are associated with less depressive symptomology than participating in rudimentary exercise alone. This highlights the importance of a multifaceted approach when trying to predict, and subsequently treat, depression in older populations.

Exercise behaviour (measured as calories per week) was tested as a predictor of depressive symptoms in the current analysis. Despite strong evidence of the antidepressive effects of exercise (Annesi, 2012; McHugh and Lawlor, 2012; Rhyner and Watts, 2016), exercise behaviour did not predict any additional variance in depressive symptoms. Latent growth modelling has demonstrated that exercise behaviour has direct and indirect pathways through mood, self-efficacy, and social support (McAuley et al., 2003). It is speculated that the indirect influences of these three psychosocial factors were able to account for all the variation in depressive symptoms. Thus, the physical characteristics of exercise, such as calorie expenditure and time spent in activities, do not appear to be important if exercise-induced mood is positive, and exercise self-efficacy and social support are maintained.

Although numerous investigations have attempted to quantify the proportion of variance that specific factors contribute to depressive symptoms, this is the first study to use an adequately powered sample to reliably examine the combination of these factors. The model that has been generated in the present study accounts for the collective strength of social support, exercise-induced mood, exercise self-efficacy, and exercise behaviour. This opens avenues for exploration to further expand the understanding of the key exercise-based components responsible for changes in depressive symptomology. Moreover, this provides insight into the multifaceted interactions between seemingly independent factors associated with depression and advances the foundations of previously established geriatric research.

### Practical Implications

Participants in the current study reported similar levels of caloric expenditure in moderate-to-high exercise (Stewart et al., 2001; Giles and Marshall, 2009), exercise self-efficacy (Resnick and Jenkins, 2000; Resnick et al., 2004), and social support (Randall et al., 2010) compared to previous studies. On the contrary, participants reported lower depressive symptoms (Ros et al., 2011; Gomez and McLaren, 2015) and more positive mood states (Huelsman et al., 1998, 2003), reflecting a tendency to report fewer negative dispositions. Nevertheless, our findings have important real-world implications for community-dwelling older adults, both as an approach to identify those susceptible to depression and/or as a precautionary intervention to manage depressive symptoms.

Depression is more difficult to treat in later life because it is often masked by other comorbid medical and psychiatric morbidities. This may include, for instance, depressed moods being confused with anxiety and other psychiatric illnesses, somatic symptoms being confused with medical illnesses, and reduction in activities and cognitive impairments being confused with ageing (Gottfries, 1998; Patel, 2001). According to the current findings, deficits in mood, self-efficacy, and social support are useful indicators for identifying those who are susceptible to depressive symptoms in older age. Thus, clinicians and researchers may be able to use these factors to identify those most at risk of depression, particularly those with comorbid psychiatric disorders or those with lower rates of treatment-seeking behaviours (Manetti et al., 2014).

Exercise programmes are a cost-effective alternative to traditional cognitive-behavioural approaches used to treat depression and have additional benefits to antidepressant medications (Mura et al., 2014). Our findings highlight the possible benefits of exercise as a behavioural intervention to target those most susceptible to depressive symptoms. Exercise can also be modified to meet the physical needs, abilities, and personal interests of aged populations, allowing exercise programmes to be personalised for the specific individuals or groups. By taking advantage of different exercise types and modalities, older adults may be able to discover specific ways to promote positive mood states, build self-efficacy beliefs, and enlist social support, therefore reducing their susceptibility to depressive symptoms.

The implementation of exercise-based intervention policies and programmes have merit to facilitate resilience to depressive symptoms during older adulthood. In particular, healthcare professionals can identify older adults at risk of negative mood states and depression, then implement group programmes that build self-efficacy and social support through regular exercise habits. Self-efficacy training has been incorporated into exercise intervention programmes to treat depressive symptoms in older adults (Resnick et al., 2008). Moreover, exercise programmes in a community and/or group setting can provide a cost-effective form of social engagement, promoting positive affective states and further enhancing self-efficacy beliefs (McAuley et al., 2000).

### Limitations and Future Directions

The current study has several limitations worthy of mention. Questionnaires in cross-sectional designs share common-method variance. For the current study, self-selection bias was observed whereby females and those with a higher level of education tended to be more likely to participate in the research and complete electronic questionnaire packages. To minimise potential bias, we used both paper and electronic questionnaires to target specific subgroups, including men’s organisations, low-income senior citizen centers, multicultural centers, and other at-risk groups.

During independent *t*-tests and χ^2^ tests, differences between paper and electronic questionnaire formats emerged for age, education, relationship status, and employment status. No significant patterns were apparent for relationship or employment status, however, younger age and higher level of education were evident. Since differences in these two demographic variables were expected (Ebert et al., 2018), we perceived this as being low risk of bias and subsequently collapsed the data into a single dataset.

It is important to note that the SPS-10, with scores ranging from 10 to 40, was used in the current study to assess participants’ social support. The histogram (see Supplementary File S1) reflected a significantly high proportion of scores falling in the upper limit of the distribution, indicating the presence of a ceiling effect. This reduced the variability in the gathered data for social support, which may falsely indicate that the factor had a smaller effect (i.e., Type II error). This is important to note when interpreting the results, as this may have underestimated the relationship between social support and other variables (Cramer and Howitt, 2004).

Although causal links have been theorised in the current study, it is important to note that this is an extension of correlational analysis, and therefore, caution should be taken when assuming any causal relationship. Instead, these findings can be used to guide the existing theories and provide a foundation for future experimental research. It is possible, for example, that (1) regular exercise induces a positive mood, which then leads to more opportunities for older adults to socialise and build support, (2) those with high social support are more likely to engage in exercise and regulate their negative mood and feelings of depression, or (3) depression levels dictate the engagement in, or avoidance of, regular exercise and social interaction during older adulthood. Randomised controlled trials and longitudinal designs in which depression and moderator/mediator variables are assessed prior to commencing an exercise-based intervention programme, then retested over one or more follow-up periods, would assist in identifying causal relationships in the regression model.

## Conclusion

Social support presents the strongest predictor of depressive symptomology, although exercise-induced mood and exercise self-efficacy have additional merit. These findings have significant implications for public health and can be used to guide exercise prescription in community-dwelling older adults. In particular, healthcare professionals may target older adults most at risk of depression and implement exercise programmes that are likely to improve mood, enhance self-efficacy, and build social support. Further investigation, however, is required to demonstrate a treatment effect.

## Data Availability Statement

The raw data supporting the conclusions of this manuscript will be made available by the authors, without undue reservation, to any qualified researcher.

## Ethics Statement

The studies involving human participants were reviewed and approved by the Human Research Ethics Committee (HREC) – Federation University Australia. Written informed consent for participation was not required for this study in accordance with the national legislation and the institutional requirements.

## Author Contributions

KM was responsible for all aspects of this project, including obtaining the ethics approval, recruiting the participants, data analysis, and the manuscript preparation. RG, CM, SM, and FG supervised the study design and assisted with the manuscript preparation. MY assisted with recruiting the participants and manuscript preparation. All authors read and approved the final manuscript.

## Conflict of Interest

The authors declare that the research was conducted in the absence of any commercial or financial relationships that could be construed as a potential conflict of interest.
