# Supplementary material for: Exercise, Mood, Self-Efficacy, and Social Support as Predictors of Depressive Symptoms in Older Adults: Direct and Interaction Effects
Source: Front Psychol. 2019 Sep 19;10:2145. doi: 10.3389/fpsyg.2019.02145 (PMC6761306; doi:10.3389/fpsyg.2019.02145)
Supplement: Supplementary file 1 [file Data_Sheet_1.PDF]

Supplementary File S1

Exercise, Mood, Self-Efficacy, and Social Support as Predictors of Depressive Symptoms in Older Adults: Direct and Interaction Effects

Kyle J. Miller[1*], Christopher Mesagno[1], Suzanne McLaren[1], Fergal Grace[1], Mark Yates[2], Rapson Gomez[1]

[1]School of Health and Life Sciences, Federation University, Ballarat, Victoria, Australia

[2]Faculty of Health, School of Medicine, Ballarat Health Services, Deakin University, Ballarat, Victoria, Australia

***Correspondence:**

Kyle J. Miller

kylemiller9@outlook.com

1. Literature Search Procedures

Two separate computerised searches were performed in the PubMed database using MeSH terms and keywords pertinent to three main concepts: age group, depression, and exercise. During the initial search, age group MeSH terms were used (i.e., 'aged', 'middle aged', 'younger adult', 'adolescent', 'child', and 'infant') in combination with depression-related MeSH terms (i.e., 'depressive disorder' and 'depression') and depression-related keywords (i.e., 'depress*' and 'dysthymi*'). A secondary search was then performed with the addition of exercise-related keywords (i.e., 'exercise', 'physical activity', 'weight training', 'weight lifting', 'resistance training', 'strength training', 'balance training', 'aerobic training', 'anaerobic training', 'yoga', 'tai chi', 'taiji', 'qigong', 'walk*', 'jog*', 'run*', 'swim*', 'danc*', and 'cycl*'). Total search results were determined based on the total number of retrieved records from inception up to and including May, 2019.

Older Adults Search Strategy #1

(aged[Mesh]) AND (depressive disorder[Mesh] OR depression[Mesh] OR depress*[Title/Abstract] OR dysthymi*[Title/Abstract])

Total Search Results = 92,108

Younger Adults Search Strategy #1

(middle aged[Mesh] OR young adult[Mesh]) AND (depressive disorder[Mesh] OR depression[Mesh] OR depress*[Title/Abstract] OR dysthymi*[Title/Abstract])

Total Search Results = 150,320

Children and Adolescents Search Strategy #1

(adolescent[Mesh] OR child[Mesh] OR infant[Mesh]) AND (depressive disorder[Mesh] OR depression[Mesh] OR depress*[Title/Abstract] OR dysthymi*[Title/Abstract])

Total Search Results = 79,308

Older Adults Search Strategy #2

(aged[Mesh]) AND (depressive disorder[Mesh] OR depression[Mesh] OR depress*[Title/Abstract] OR dysthymi*[Title/Abstract]) AND (exercise[Title/Abstract] OR physical activity[Title/Abstract] OR weight training[Title/Abstract] OR weight lifting[Title/Abstract] OR resistance training[Title/Abstract] OR strength training[Title/Abstract] OR balance training[Title/Abstract] OR aerobic training[Title/Abstract] OR anaerobic training[Title/Abstract] OR yoga[Title/Abstract] OR tai chi[Title/Abstract] OR taiji[Title/Abstract] OR qigong[Title/Abstract] OR walk*[Title/Abstract] OR jog*[Title/Abstract] OR run*[Title/Abstract] OR swim*[Title/Abstract] OR danc*[Title/Abstract] OR cycl*[Title/Abstract])

Total Search Results = 8,113

Younger Adults Search Strategy #2

(middle aged[Mesh] OR young adult[Mesh]) AND (depressive disorder[Mesh] OR depression[Mesh] OR depress*[Title/Abstract] OR dysthymi*[Title/Abstract]) AND (exercise[Title/Abstract] OR physical activity[Title/Abstract] OR weight training[Title/Abstract] OR weight lifting[Title/Abstract] OR resistance training[Title/Abstract] OR strength training[Title/Abstract] OR balance training[Title/Abstract] OR aerobic training[Title/Abstract] OR anaerobic training[Title/Abstract] OR yoga[Title/Abstract] OR tai chi[Title/Abstract] OR taiji[Title/Abstract] OR qigong[Title/Abstract] OR walk*[Title/Abstract] OR jog*[Title/Abstract] OR run*[Title/Abstract] OR swim*[Title/Abstract] OR danc*[Title/Abstract] OR cycl*[Title/Abstract])

Total Search Results = 11,919

Children and Adolescents Search Strategy #2

(adolescent[Mesh] OR child[Mesh] OR infant[Mesh]) AND (depressive disorder[Mesh] OR depression[Mesh] OR depress*[Title/Abstract] OR dysthymi*[Title/Abstract]) AND (exercise[Title/Abstract] OR physical activity[Title/Abstract] OR weight training[Title/Abstract] OR weight lifting[Title/Abstract] OR resistance training[Title/Abstract] OR strength training[Title/Abstract] OR balance training[Title/Abstract] OR aerobic training[Title/Abstract] OR anaerobic training[Title/Abstract] OR yoga[Title/Abstract] OR tai chi[Title/Abstract] OR taiji[Title/Abstract] OR qigong[Title/Abstract] OR walk*[Title/Abstract] OR jog*[Title/Abstract] OR run*[Title/Abstract] OR swim*[Title/Abstract] OR danc*[Title/Abstract] OR cycl*[Title/Abstract])

Total Search Results = 3,319

2. Histogram of Social Support

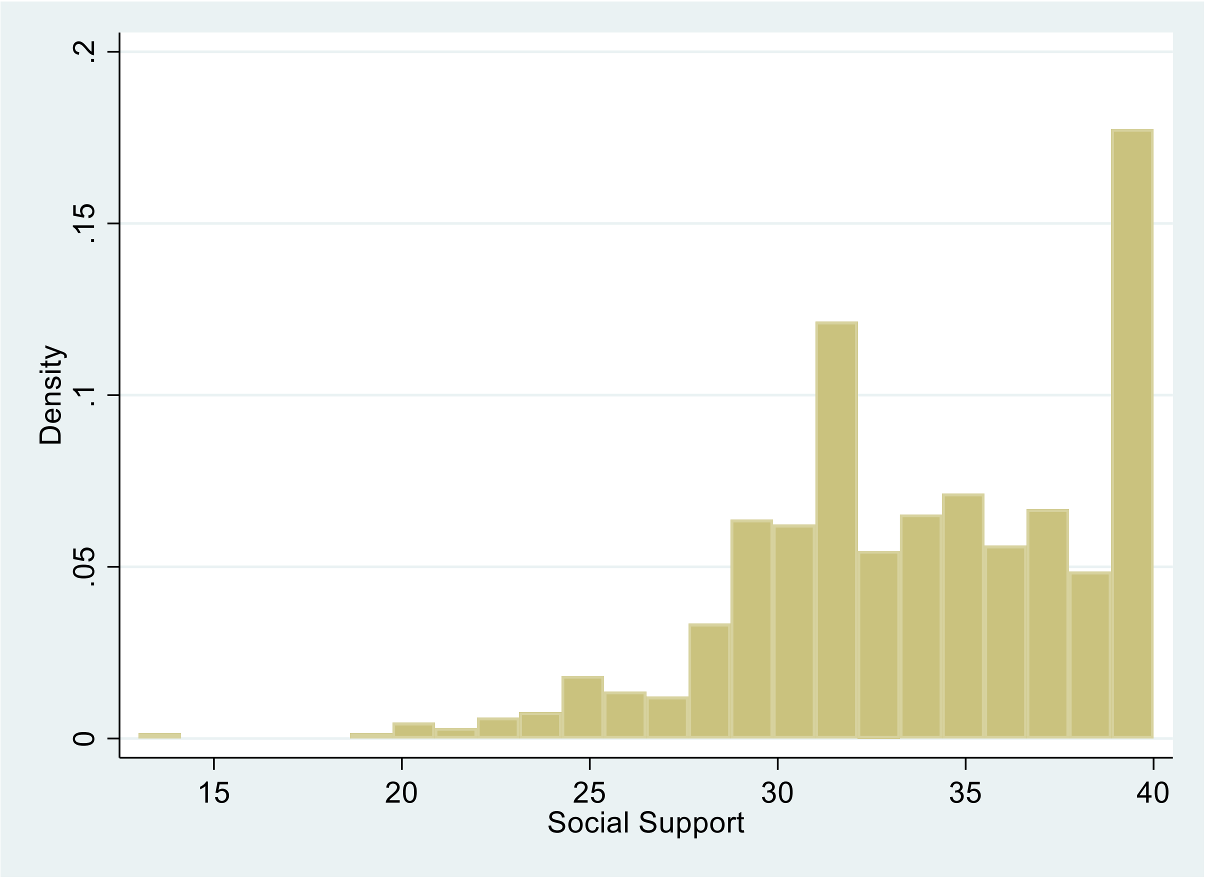

Figure S1. Histogram depicting the distribution of social support scores measured with the Social Provisions Scale - Short Form (SPS-10) in community-dwelling older adults aged 65-96 years (*n* = 586).
